# Supplementary material for: Plant Viruses Can Alter Aphid-Triggered Calcium Elevations in Infected Leaves
Source: Cells. 2021 Dec 14;10(12):3534. doi: 10.3390/cells10123534 (PMC8700420; doi:10.3390/cells10123534)
Supplement: Supplementary file 1 [file cells-10-03534-s001.zip › Suppplementary_Figure_S1.pdf]

Supplemental Figure S1

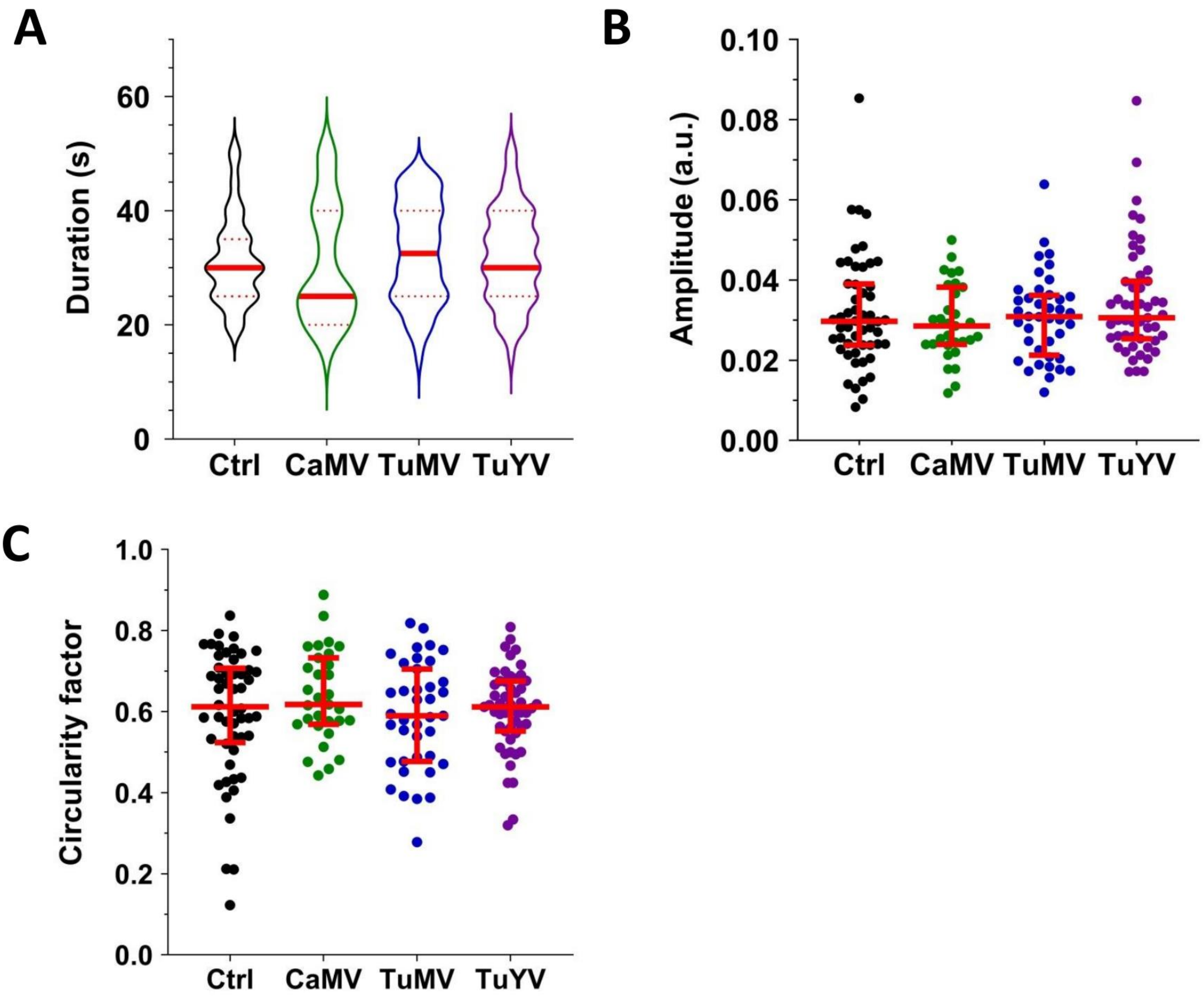

Other properties of aphid-triggered calcium waves. The duration of the calcium propagations (A), their amplitudes (B) and their circularity factors (C) were plotted. None of the parameters was significantly different in infected versus healthy control plants. The plots show medians and quartiles. The ends of the whiskers present lowest and highest datum still within 1.5 IQR of the lower and higher quartile, respectively.
